# Supplementary material for: MicroED as a powerful tool for structure determination of macrocyclic drug compounds directly from their powder formulations
Source: bioRxiv. 2023 Aug 1:2023.07.31.551405. Preprint. [Version 1] doi: 10.1101/2023.07.31.551405 (PMC10418104; doi:10.1101/2023.07.31.551405)
Supplement: Supplement 1 [file media-1.pdf]

SUPPLEMENTARY INFORMATION

MicroED as a powerful tool for structure determination of macrocyclic drug compounds directly from their powder formulations

Danelius E[1,2*]., Bu G[2*], Wieske H[3] and Gonen T[1,2,4$]

1. Howard Hughes Medical Institute, University of California Los Angeles, Los Angeles, CA 90095, USA.
2. Department of Biological Chemistry, University of California Los Angeles, 615 Charles E.Young Drive South, Los Angeles, CA 90095, USA.
3. Department of Chemistry – BMC, Uppsala University, Husargatan 3, 75237 Uppsala, Sweden.
4. Department of Physiology, University of California Los Angeles, 615 Charles E. Young Drive South, Los Angeles, CA 90095, USA.

* Denotes equal contribution

$ Correspondence to T.G tgonen@g.ucla.edu

Supplementary Data Table 1. MicroED data collection and refinement statistics for pacritinib.

Stoichiometric formula	$C_{28}H_{32}N_4O_3$
Radiation wavelength (Å)	0.0251
Temperature (K)	80
Number of crystals	1
Crystal description	plate
Resolution (Å)	0.62
Crystal system	monoclinic
Space group	Pc
a (Å)	10.52
b (Å)	14.48
c (Å)	15.90
α (°)	90
β (°)	92.944
ɣ (°)	90
Z	4
Total reflections	28,160
Unique reflections	9,857
R_{obs} (%)	9.1
R_{meas} (%)	11.2
I/σI	6.17
$CC_{1/2}$ (%)	99.5
Completeness (%)	89.9
R_1	0.1537
wR_2	0.4132
GooF	1.162
CCDC number	

Supplementary Data Table 2. MicroED data collection and refinement statistics for romidepsin.

Stoichiometric formula	$C_{24}H_{36}N_4O_6S_2$
Radiation wavelength (Å)	0.0251
Temperature (K)	80
Number of crystals	8
Crystal description	plate
Resolution (Å)	0.80
Crystal system	monoclinic
Space group	$P2_1$
a (Å)	9.17
b (Å)	16.58
c (Å)	9.61
α (°)	90
β (°)	92.952
ɣ (°)	90
Z	2
Total reflections	11,059
Unique reflections	2,513
R_{obs} (%)	23.8
R_{meas} (%)	26.6
I/σI	4.97
$CC_{1/2}$ (%)	98.1
Completeness (%)	81.0
R_1	0.2293
wR_2	0.4488
GooF	1.248
CCDC number	

Supplementary Data Table 3. MicroED data collection and refinement statistics for simeprevir.

Stoichiometric formula	$C_{38}H_{47}N_5O_7S_2$
Radiation wavelength (Å)	0.0251
Temperature (K)	80
Number of crystals	8
Crystal description	needle
Resolution (Å)	0.85
Crystal system	triclinic
Space group	P1
a (Å)	5.08
b (Å)	18.69
c (Å)	19.74
α (°)	89.182
β (°)	86.455
ɣ (°)	97.320
Z	2
Total reflections	22,621
Unique reflections	5,294
R_{obs} (%)	16.0
R_{meas} (%)	18.2
I/σI	5.43
$CC_{1/2}$ (%)	98.4
Completeness (%)	84.2
R_1	0.1324
wR_2	0.3424
GooF	1.030
CCDC number	

Supplementary Data Table 4. MicroED data collection and refinement statistics for brefeldin A.

Stoichiometric formula	$C_{16}H_{24}O_4$
Radiation wavelength (Å)	0.0251
Temperature (K)	80
Number of crystals	4
Crystal description	plate
Resolution (Å)	0.85
Crystal system	orthorhombic
Space group	$P2_12_12_1$
a (Å)	7.51
b (Å)	11.00
c (Å)	19.05
α (°)	90
β (°)	90
ɣ (°)	90
Z	4
Total reflections	10,217
Unique reflections	1,471
R_{obs} (%)	17.2
R_{meas} (%)	18.6
I/σI	7.68
$CC_{1/2}$ (%)	98.8
Completeness (%)	92.9
R_1	0.1229
wR_2	0.3368
GooF	1.200
CCDC number	

Supplementary Data Table 5. MicroED data collection and refinement statistics for paritaprevir.

Stoichiometric formula	$C_{40}H_{43}N_7O_7S$
Radiation wavelength (Å)	0.0251
Temperature (K)	80
Number of crystals	2
Crystal description	needle
Resolution (Å)	0.85
Crystal system	orthorhombic
Space group	$P2_12_12_1$
a (Å)	5.09
b (Å)	15.61
c (Å)	50.78
α (°)	90
β (°)	90
ɣ (°)	90
Z	4
Total reflections	15,086
Unique reflections	3,607
R_{obs} (%)	20.2
R_{meas} (%)	23.2
I/σI	5.20
$CC_{1/2}$ (%)	98.9
Completeness (%)	89.0
R_1	0.1472
wR_2	0.4080
GooF	1.156
CCDC number	

Supplementary Data Table 6. MicroED data collection and refinement statistics for troleandomycin.

Stoichiometric formula	$C_{41}H_{67}NO_{15}$
Radiation wavelength (Å)	0.0251
Temperature (K)	80
Number of crystals	1
Crystal description	needle
Resolution (Å)	1.70
Crystal system	orthorhombic
Space group	$P2_12_12_1$
a (Å)	8.69
b (Å)	23.06
c (Å)	47.33
α (°)	90
β (°)	90
ɣ (°)	90
Z	4
Total reflections	3,162
Unique reflections	1,011
R_{obs} (%)	12.1
R_{meas} (%)	16.5
I/σI	6.3
$CC_{1/2}$ (%)	98.3
Completeness (%)	85.5
R_{work}	0.26
R_{free}	0.27

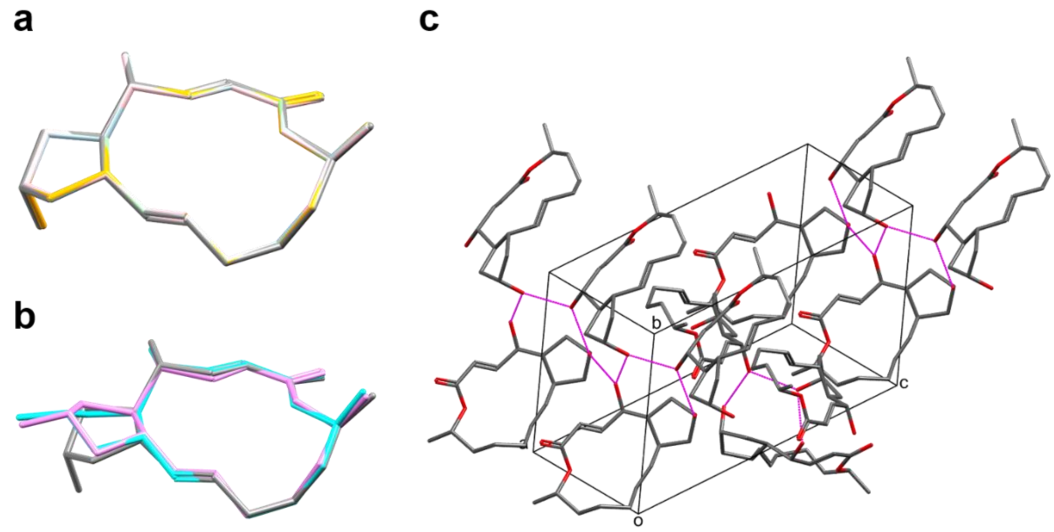

Figure SI-1. Structure and packing of brefeldin A. a. Overlay of the heavy atoms of the macrocyclic rings of the MicroED structure (gray) with the SCXRD structures: CCDC codes BREFEL[1] (pink, rmsd=0.0509 Å), BREFEL02[2] (orange, rmsd=0.0384 Å), BREFEL03[3] (light green, rmsd=0.0394 Å), and BREFEL04[4] (light blue, rmsd=0.0448 Å). **b.** Overlay of the heavy atoms of the macrocyclic rings of the MicroED structure (gray) with the target bound structures: PDB codes 1RE0[5] (cyan, rmsd=0.106 Å), and 1R8Q[6] (violet, rmsd=0.114 Å). **c.** Crystal packing of brefeldin A with the unit cell box shown in black. Atom colors: C, gray; O, red. Intermolecular hydrogen bonds (1.68 Å and 1.75 Å) are shown in magenta. Hydrogen atoms are omitted for clarity.

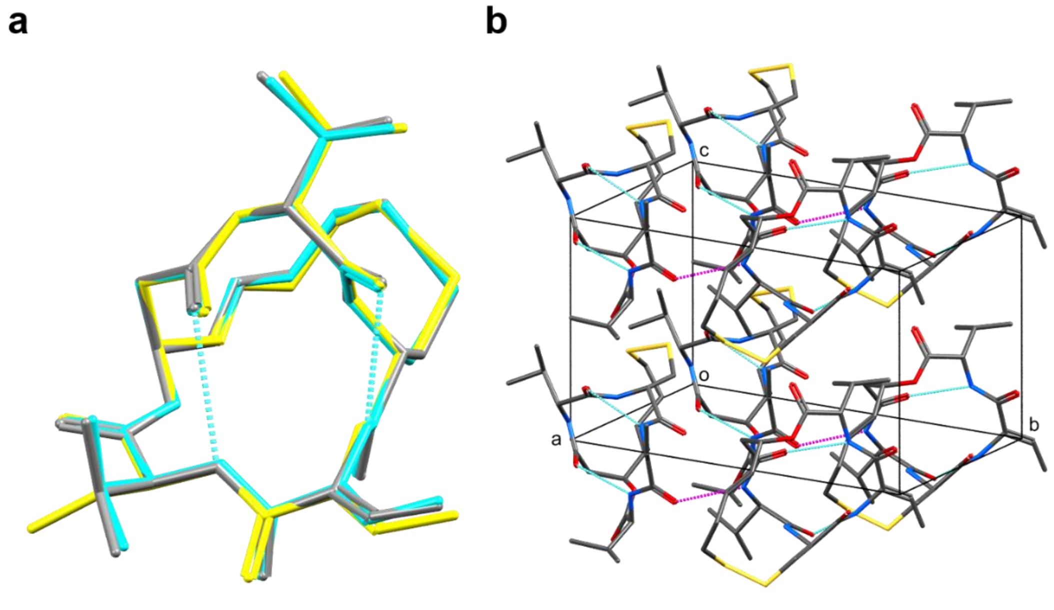

Figure SI-2. Structure and packing of romidepsin. a. Overlay of the heavy atoms of the macrocyclic rings of the MicroED structure (gray) with the SCXRD structures: CCDC codes QEDJOA[7] (cyan, rmsd=0.0977 Å) and LIBDEF[8] (yellow, rmsd=0.143 Å). Intramolecular hydrogen bonds (2.16 Å and 2.36 Å) are shown in cyan dashed lines. **b.** Crystal packing of romidepsin with the unit cell box is shown in black. Atom colors: C, gray; N, blue; O, red; S, yellow. Intramolecular hydrogen bonds (2.16 Å and 2.36 Å) are shown in cyan dashed lines. Intermolecular hydrogen bonds (2.21 Å) are shown in magenta. Hydrogen atoms are omitted for clarity.

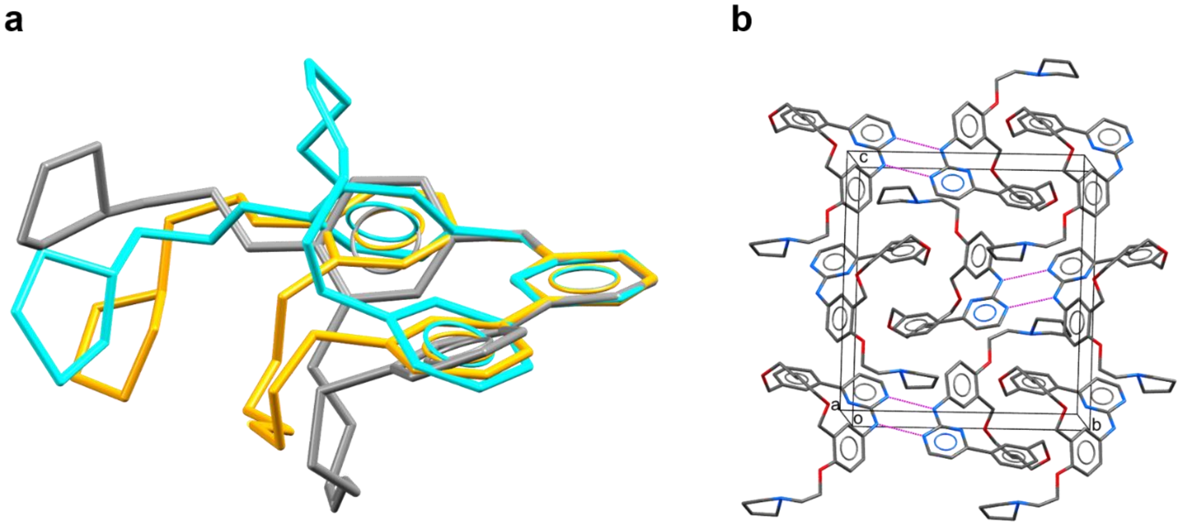

Figure SI-3. Structure and packing of pacritinib. a. Overlay of the biphenyl aromatic atoms of the macrocyclic rings of the two MicroED conformations (conformer 1, gray; conformer 2, cyan; rmsd=1.51 Å when comparing macrocyclic heteroatoms) and the target bound structure (PDB ID 5LBZ[9] orange, rmsd=0.365 Å and 1.41 Å compared with conformer 1 and 2, respectively). **b.** Crystal packing of pacritinib with the unit cell box is shown in black. Atom colors: C, gray; N, blue; O, red. Intermolecular hydrogen bonds (1.90 Å and 2.10 Å) are shown in magenta. Hydrogen atoms are omitted for clarity.

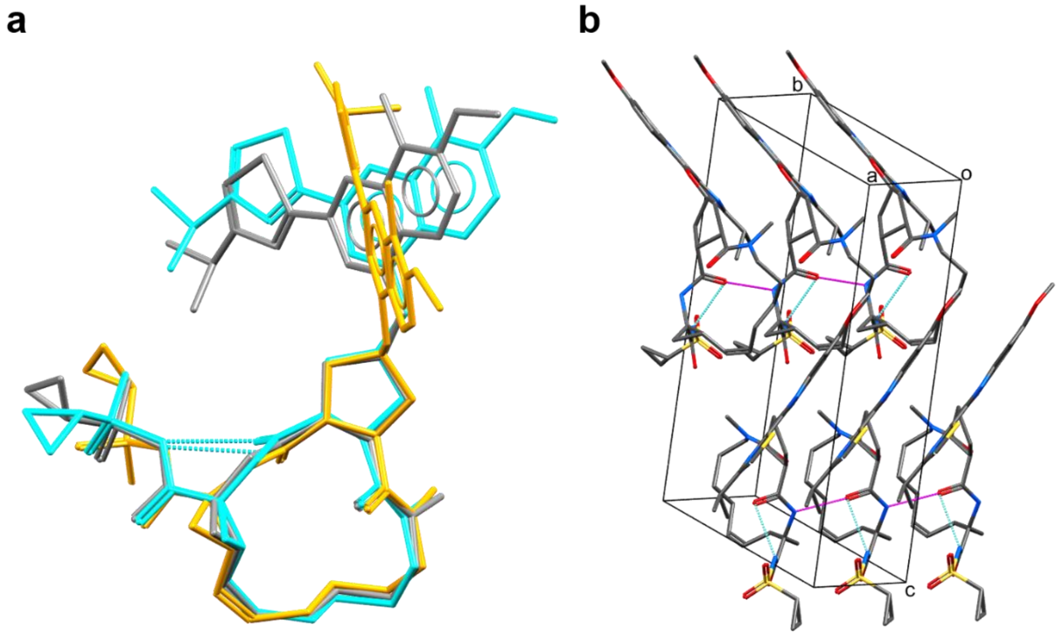

Figure SI-4. Structure and packing of simeprevir. a. Overlay of the heavy atoms of the macrocyclic rings of the two MicroED conformations (conformer 1, gray; conformer 2, cyan; rmsd=0.196 Å) and the target bound structure (PDB ID: 3KEE[10], orange, rmsd=0.264 Å and 0.406 Å compared with conformer 1 and 2, respectively). Intramolecular hydrogen bonds (2.05 Å conformer 1, and 2.29 Å conformer 2) shown in cyan dashed lines. **b.** Crystal packing of simeprevir with the unit cell box is shown in black. Atom colors: C, gray; N, blue; O, red; S, yellow. Intramolecular hydrogen bonds (2.05 Å conformer 1, and 2.29 Å conformer 2) are shown in cyan dashed lines. Intermolecular hydrogen bonds (2.29 Å conformer 1, and 2.24 Å conformer 2) are shown in magenta. Hydrogen atoms are omitted for clarity.

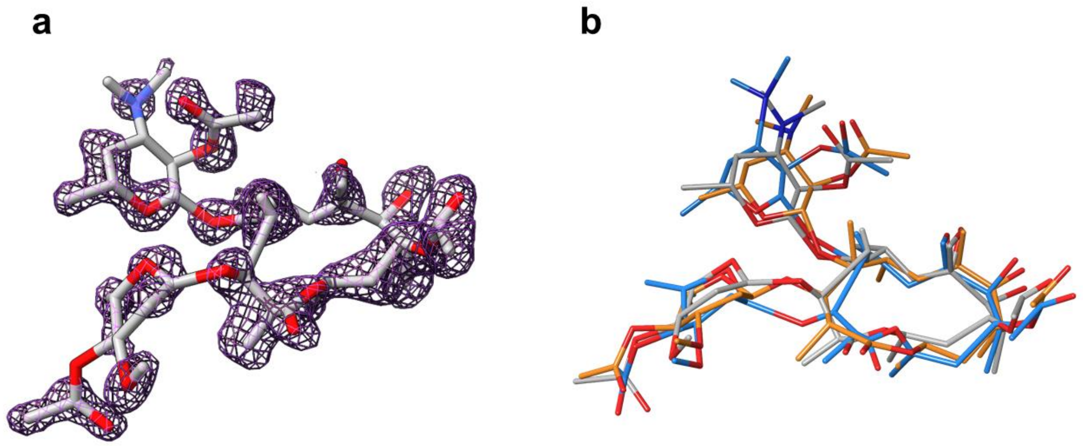

Figure SI-5. Structure of troleandomycin. a. MicroED structure with the 2Fo-Fc map in grey mesh (2σ). Two components were found in the molecular replacement, conformer 1 shown here. Atom colors: C, gray; N, blue; O, red. Hydrogen atoms are omitted for clarity. **b.** Overlay of the heavy atoms of the macrocyclic rings of the two MicroED conformations (conformer 1, gray; conformer 2, blue) and the target bound structure (PDB ID: 1OND[11], orange).

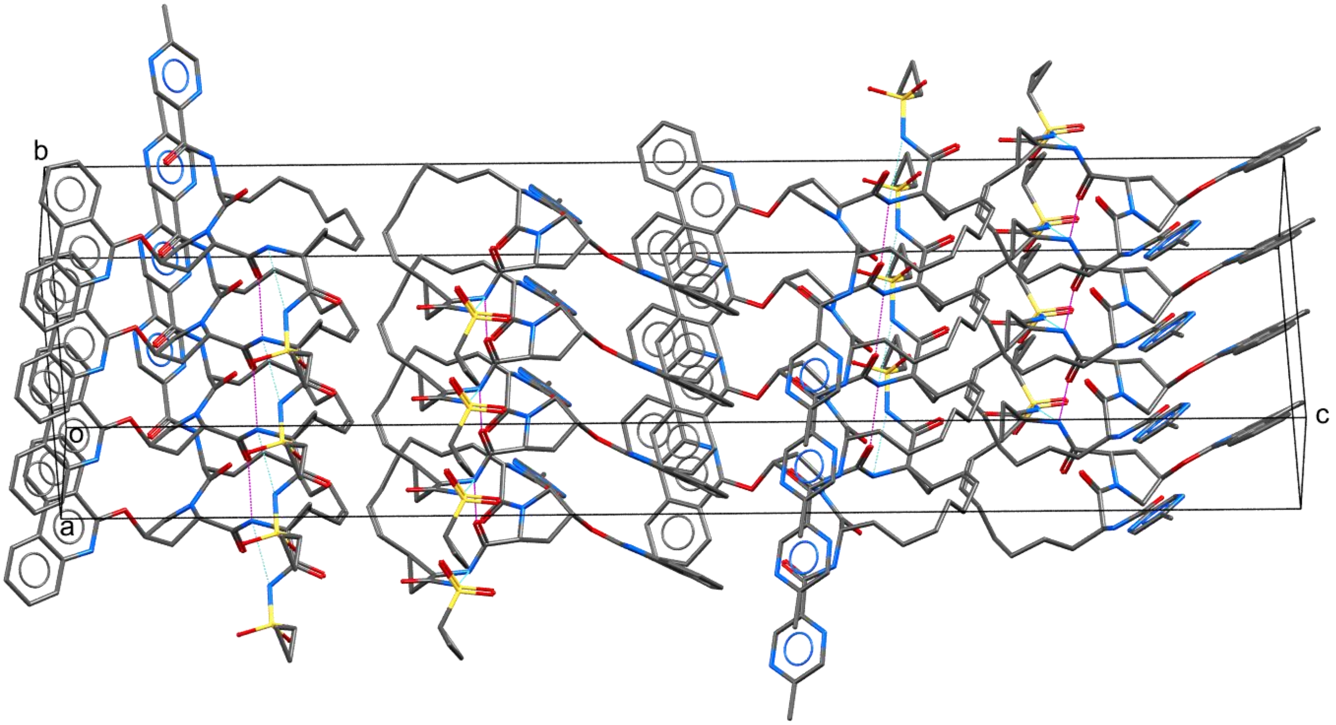

Figure SI-6. Packing of paritaprevir. Crystal packing of paritaprevir with the unit cell box is shown in black. Atom colors: C, gray; N, blue; O, red; S, yellow. Intramolecular hydrogen bonds (2.19 Å) are shown in cyan dashed lines, and intermolecular hydrogen bonds (2.26 Å) are shown in magenta. Hydrogen atoms are omitted for clarity.

References

1. Weber H.P., Hauser D., Sigg H.P. Structure of brefeldin A. *Helvetica Chimica Acta* **1971** *54*(8): 2763–2766.
2. Fuchs M., Fürstner A., Trans-Hydrogenation: Application to a Concise and Scalable Synthesis of Brefeldin A. *Angewandte Chemie International Edition.* **2015** *54*(13): 3978–3982.
3. Huang H., Liu T., Guo J., Yu L., Wu X., He Y., Li D., Liu J., Zhang K., Zheng X., Goodin S. Brefeldin A enhances docetaxel-induced growth inhibition and apoptosis in prostate cancer cells in monolayer and 3D cultures. *Bioorganic & Medicinal Chemistry Letters.* **2017** *27*(11): 2286–2291.
4. Zeng F., Chen C., Chnani A., *et al*. Dibrefeldins A and B, A pair of epimers representing the first brefeldin A dimers with cytotoxic activities from Penicillium janthinellum. *Bioorganic Chemistry.* **2019** *86*: 176–182.
5. Mossessova E., Corpina R.A., Goldberg J. Crystal structure of ARF1• Sec7 complexed with Brefeldin A and its implications for the guanine nucleotide exchange mechanism. *Molecular Cell*. **2003** *12*(6): 1403–1411.
6. Renault L., Guibert B., Cherfils J. Structural snapshots of the mechanism and inhibition of a guanine nucleotide exchange factor. *Nature* **2003** *426*(6966): 525–530.
7. Liu X.Y., Wang C., Cheng Y.Q. FK228 from Burkholderia thailandensis MSMB43. *Acta Crystallographica Section E* **2012** *68*(9): 2757–2758.
8. Shigematsu N., Ueda H., Takase S., Tanaka H., Yamamoto K., Tada T. FR901228, a novel antitumor bicyclic depsipeptide produced by Chromobacterium violaceum No. 968 II. Structure determination. *The Journal of Antibiotics* **1994** *47*(3): 311–314.
9. Klaeger S., Heinzlmeir S., Wilhelm M., *et al*. The target landscape of clinical kinase drugs. *Science* **2017** *358*(6367): eaan4368.
10. Cummings M.D., Lindberg J., Lin TI., *et al*. Induced-Fit Binding of the Macrocyclic Noncovalent Inhibitor TMC435 to its HCV NS3/NS4A Protease Target. *Angewandte Chemie International Edition* **2010** *49*(9): 1652–1655.
11. Berisio R., Schluenzen F., Harms J. *et al*. Structural insight into the role of the ribosomal tunnel in cellular regulation. *Nature Structural and Molecular Biology* **2003** *10*: 366–370.
